# Early-life DNA methylation profiles are indicative of age-related transcriptome changes

**DOI:** 10.1186/s13072-019-0306-5

**Published:** 2019-10-08

**Authors:** Niran Hadad, Dustin R. Masser, Laura Blanco-Berdugo, David R. Stanford, Willard M. Freeman

**Affiliations:** 1 Oklahoma Center for Neuroscience, Oklahoma City, OK USA; 2 Reynolds Oklahoma Center on Aging, SLY-BRC 1370, 975 NE 10th St, Oklahoma City, OK 73104 USA; 3 Department of Physiology, Oklahoma City, OK USA; 4 The Jackson Laboratory, Bar Harbor, ME 04609 USA; 5 Oklahoma Nathan Shock Center, University of Oklahoma Health Sciences Center, Oklahoma City, OK USA; 6 Oklahoma City VA Medical Center, Oklahoma City, OK 73104 USA; 7 Present Address: The Jackson Laboratory, Bar Harbor, ME 04609 USA

**Keywords:** DNA methylation, Aging, Epigenetics, Gene regulation, Hippocampus

## Abstract

**Background:**

Alterations to cellular and molecular programs with brain aging result in cognitive impairment and susceptibility to neurodegenerative disease. Changes in DNA methylation patterns, an epigenetic modification required for various CNS functions are observed with brain aging and can be prevented by anti-aging interventions, but the relationship of altered methylation to gene expression is poorly understood.

**Results:**

Paired analysis of the hippocampal methylome and transcriptome with aging of male and female mice demonstrates that age-related differences in methylation and gene expression are anti-correlated within gene bodies and enhancers. Altered promoter methylation with aging was found to be generally un-related to altered gene expression. A more striking relationship was found between methylation levels at young age and differential gene expression with aging. Highly methylated gene bodies and promoters in early life were associated with age-related increases in gene expression even in the absence of significant methylation changes with aging. As well, low levels of methylation in early life were correlated to decreased expression with aging. This relationship was also observed in genes altered in two mouse Alzheimer’s models.

**Conclusion:**

DNA methylation patterns established in youth, in combination with other epigenetic marks, were able to accurately predict changes in transcript trajectories with aging. These findings are consistent with the developmental origins of disease hypothesis and indicate that epigenetic variability in early life may explain differences in aging trajectories and age-related disease.

## Introduction

Epigenetic modifications, chromatin, and direct DNA modifications are key genomic regulatory processes required for proper development [1], gene imprinting [2–4], X chromosome inactivation [5–7], gene expression regulation [8], and genomic organization [9–11]. Disruptions to the epigenome can alter basic cellular regulation leading to a wide range of dysfunctional molecular programs [10–12]. Dysregulated epigenetic control with aging has been proposed as an etiological factor common to age-related diseases ranging from diabetes to neurodegenerative diseases such as Alzheimer’s disease [13–18]. DNA methylation has been widely studied in geroscience research as methylation at specific loci is indicative of chronological age [19–22] and can potentially be an indicator of ‘biological’ aging [23, 24]. DNA methylation primarily occurs in a CpG context; however, non-CpG methylation is abundant in the central nervous system (CNS) [1, 25] and has only been minimally examined with aging [26, 27]. With the growing understanding that DNA methylation is dynamic, the role of alterations in DNA methylation patterns in regulating gene expression changes during development, aging, and disease is of particular interest.

DNA methylation changes with aging demonstrate both tissue specificity and conservation across tissues depending on the specific genomic location [28–30]. Conserved changes with aging across tissues in the form of epigenetic clocks have proved to be a powerful tool for estimating chronological age and are predictive of all-cause mortality [24, 31, 32]. Tissue-specific DNA methylation changes with aging on the other hand may underlie organ/cell-specific deficits. For example, in the liver, gene body hypermethylation occurs primarily in genes involved in lipid metabolism [33], while in the brain age-related methylation changes occur in genes involved in synaptic transmission and cellular integrity [26]. It is important to note that changes in methylation also occur in pathways implicated to be dysregulated with aging systemically, such as the insulin-signaling pathway and cellular senescence [34–37]. Recent studies show that age-related DNA methylation changes in blood [38, 39], kidney [40], liver [33, 37], and the hippocampus [26], can be partially prevented by dietary, genetic, and pharmacological pro-longevity interventions providing further support for the association between DNA methylation and aging.

In the CNS, DNA methylation plays an important role in cellular differentiation [41–43], synaptic formation and function [44, 45], and in molecular mechanisms underlying learning and memory formation [46]. These processes are known to be impaired with aging [47]; however, whether age-related methylation differences contribute to the decline of these processes is unknown. Global levels of DNA methylation have been proposed to decrease with aging [48], but this has not been observed in brain samples using modern sequencing techniques [49, 50]. Rather specific loci in the genome undergo hypermethylation and hypomethylation with aging [27]. In addition to differences in methylation, with aging there is increased variability in CpG methylation [51]. Similar findings are observed in Alzheimer’s disease (AD) patients, specifically in genes directly linked to AD [17]. Thus, epigenetic mechanisms may contribute to age-related impairments and disease through altering gene expression, but little is known about the effects of age-related changes in methylation on gene expression regulation in the brain. Understanding the role age-related differential methylation plays in brain aging may allow for identification of regulatory processes contributing to the development of neuropathologies.

In previous studies we have characterized changes in methylation and transcription with aging in the hippocampus of male and female mice, finding a core of sex common changes with the majority of age-related changes being sexually divergent [27, 52]. Here we sought to understand the effect of age-related differential methylation on gene expression using paired DNA methylation, by whole-genome bisulfite sequencing (WGBS), and transcriptome, by RNA-sequencing, data from the same samples. We find that differential methylation in gene body and enhancer elements inversely correlates with aging gene expression. This relationship is generally weak and accounts for a small fraction of the differentially expressed genes with aging. A stronger correlation was observed between age-related differential gene expression and early-life promoter and gene body methylation patterns, an association that was independent of age-related differential methylation. Furthermore, DNA methylation levels were able to predict whether transcriptional changes with age will undergo up- or downregulation with aging. The predictive ability increased when combined with other epigenetic marks. The broad implication of our findings is that early programming of the epigenome during development and/or early adulthood may impact transcriptional trajectories late in life. Understanding epigenetic differences that occur during development may help explain late-life molecular responses in the CNS and possibly differences in susceptibility to adverse conditions between individuals.

## Results

### Characterization of differential methylation in the hippocampus using whole-genome bisulfite sequencing

To assess the relationship between hippocampal age-related differential methylation and age-related transcriptional changes we first analyzed differential methylation with aging using WGBS in both male and female mice. Previous studies characterizing differential methylation in the hippocampus with aging focused on just global levels of methylation or used approaches that allowed for high-resolution analysis of portions (~ 10%) of the genome [27, 49]. Whole-genome bisulfite sequencing provides the most comprehensive analysis of gene methylation by covering the majority of CpG sites across the genome. Sequencing methods that examine smaller portions of the genomic CpG sites provide a limited and incomplete view of genic methylation (Additional file 1: Figure S1).

The average methylation level across all CpGs in young (3 months) and old (24 months) animals demonstrate no differences with aging (FY 74% ± 0.2, FO 73.5% ± 0.4, MY 74.1% ± 0.5, MO 72.5% ± 1.4, Additional file 2: Figure S2). Similarly, no difference in transposable element CpG methylation with age was evident. No differences in average methylation levels between males and females were observed. These agree with previous findings that there is no hypomethylation with aging in the murine hippocampus [49, 50].

**Fig. 1:**
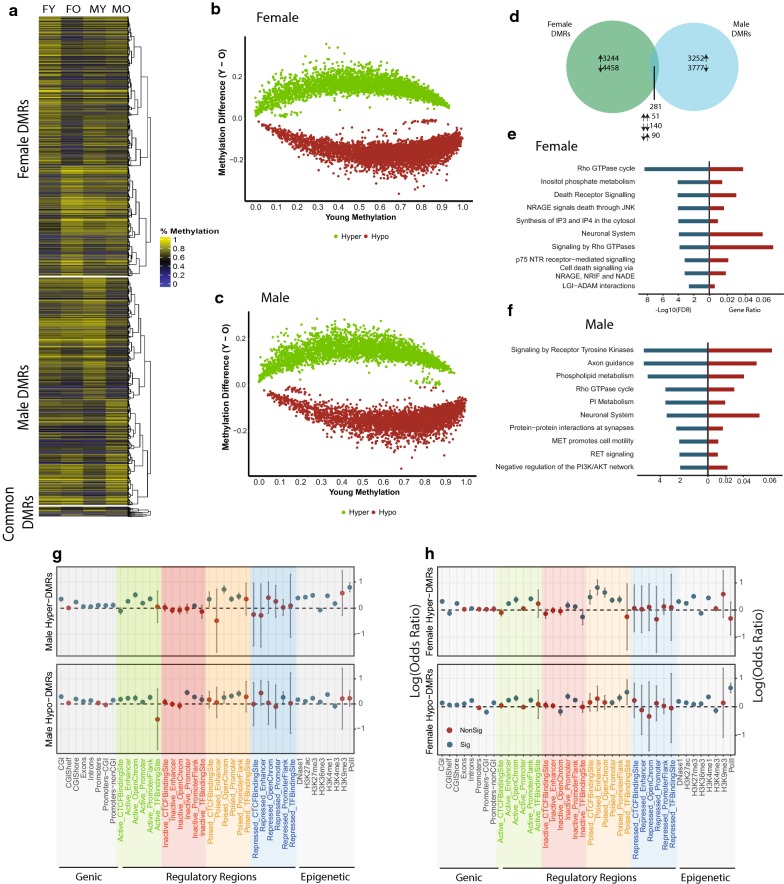
Whole-genome analysis of age-related differential methylation in males and females. **a** Heatmap of age-related differentially methylated regions, age-DMR (Fisher Exact Test with FDR < 0.05, *n* = 3/group) across all groups. Dot plot showing changes in methylation with aging relative to baseline methylation in young animals in males (**b**) and females (**c**).** d** Overlap between age-DMRs in males and in females and the directionality of methylation changes of common age-DMRs. Pathway enrichment of genes containing age-DMR within their gene body in females (**e**) and in males (**f**). Significant enrichment was determined by hypergeometric test (*p* < 0.05). **g**, **h** Over- and under-representation of age-DMRs in genic regions, CpG islands, and regulatory elements in the brain divided by their activation state, and regulatory elements annotated by specific histone marks in males and females. Over- and under-representation were determined using hypergeometric test (*p* < 0.05)

To determine regions of differential methylation, the genome was binned to 500 bp non-overlapping windows. Windows with ≥ 10 CpGs and at least 3× coverage per CpG were retained yielding 979,603 regions analyzed for differential methylation with aging. Both males and females had roughly similar numbers of age-related differentially methylated regions (age-DMRs: 7702 in females vs 7029 in males) and showed a slight bias towards hypomethylation (Fig. 1a–d). Only 2% of all age-DMRs were common to both males and females (Fig. 1b). Of these sex-common changes, 68% were commonly regulated, e.g., hypermethylated in both males and females (*χ*^2^ test of independence *p* value = 1.3 × 10^−6^). These results demonstrate that genome-wide, age-related changes in DNA methylation are predominately sex-specific, in agreement with prior findings [27].

Functional enrichment of genes containing age-DMRs revealed that although age-DMRs in males and females occurred in different genomic locations, genes containing age-related differential methylation are enriched in pathways with functional similarities, for example, genes containing age-DMRs in females are enriched in inositol phosphate metabolism, while genes containing age-DMRs in males are enriched in phospholipid metabolism and phosphoinositol metabolism (Fig. 1e, f, Additional file 3: Table S1, Additional file 4: Table S2). Generally, pathways common to both males and females are involved in glucose and lipid metabolism, neuronal interactions, and cellular integrity. These results suggest that while sex-divergence occurs at the level of the genome, the pathways affected by aging may still be functionally similar.

Age-DMRs were assessed for their enrichment across genomic features and gene regulatory elements. Over-representation of age-DMRs was observed in CpG islands and shelves, and within gene bodies (Fig. 1g, h). Generally, DMRs were not enriched in promoter regions, but when separated according whether the promoter contained a CpG island, significant enrichment of age-DMRs is observed in promoters without a CpG island. This is consistent with previous studies indicating that methylation of promoter CpG islands generally does not change with aging [53, 54]. Age-DMRs were over-represented in active and poised distal gene regulatory regions, namely active enhancers and promoter flanks. This was also evident by enrichment of age-DMRs in hippocampal H3K27ac and H3K4me1 peaks, both indicators of active and poised enhancers [55, 56] (Fig. 1e). Hypomethylated age-DMRs were also over-represented in H3K36me3, a marker of exons and transcriptional elongation [57, 58] shown to be altered with aging and associated with longevity [59, 60], and in H3K27me3, a marker associated with gene repression (Fig. 1g, h). Overall, enrichment of age-DMRs in genomic regions suggest that methylation of certain genomic regions is more susceptible to change with age as compared with others.

### Association between differential gene expression and differential methylation with aging

**Fig. 2:**
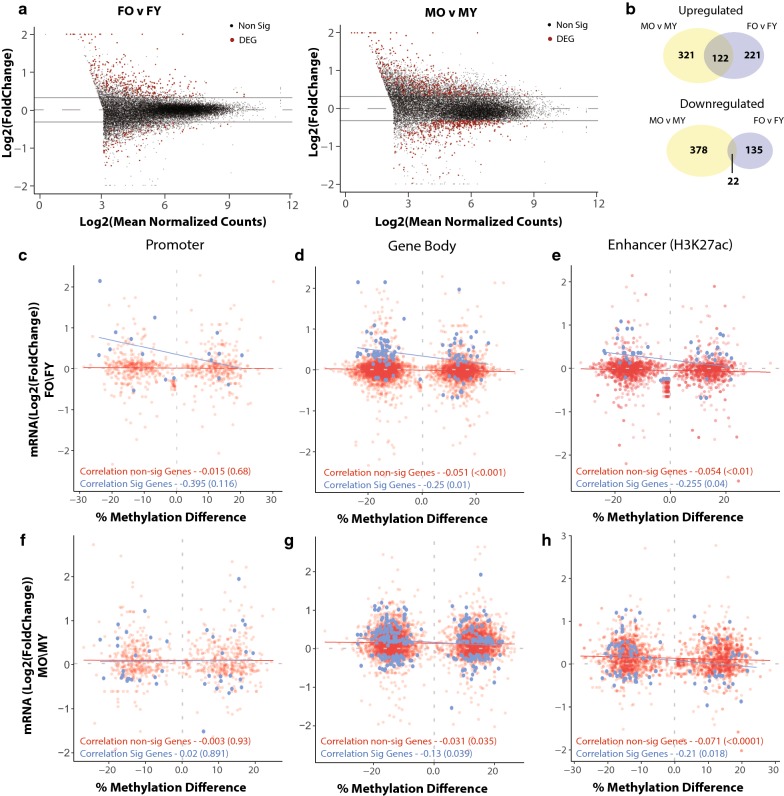
Differential methylation with aging is anti-correlated with expression changes in gene body and enhancer regions. **a** Volcano plots of mRNA differential expression with aging (multiple linear regression, FDR < 0.05, |FC| > 1.25, *n* = 6/group) in males and females. **b** Venn diagrams of the overlap of upregulated and downregulated differentially expressed genes between males and females. Correlation between age-DMRs mapped to promoters (**c**, **f**), gene body (D,G) or enhancer regions (**e**, **h**) and gene expression fold change (O/Y) in statistically significant (blue) and non-statistically significant genes (red) in females (**c**–**e**) and males (**f**–**h**)

DNA methylation functions to modulate genomic architecture and regulate gene expression. However, the relationship of differential methylation to altered steady state gene expression with aging has not been comprehensively addressed. We used RNA-sequencing to analyze transcriptional differences with aging in the same samples used for methylation analysis and correlated age-DMRs with age-related differentially expressed genes (age-DEGs) in the hippocampus. With aging 781 genes were differentially expressed with aging in males and 433 in females (multiple linear regressions, fdr < 0.05 and |FC| > 1.25) (Fig. 2a, b). Approximately 1/3 of the genes upregulated with aging were common between males and females (Fig. 2b), and only 22 downregulated genes were common between the sexes (*χ*^2^ test of independence *p* value < 2.2 × 10^−16^). This is consistent with previous findings reporting sexual divergence in transcriptional profiles in addition to a common core set of genes with aging [52].

In both males and females, only a small number of age-DEGs contained an age-DMR in their promoter region (± 1 kb of the TSS). The association between age-DMRs and differentially expressed genes with aging in promoters was not significant in both males and females (Fig. 2c, f). When assessing all age-DMRs independent of their location in the gene body (TSS to TES), a weak negative correlation is observed in both males (*r* = − 0.13, *p* = 0.039) and females (*r* = − 0.25, *p* = 0.01) (Fig. 2d, g). On average, differentially expressed genes and those not changing in expression with aging had similar methylation values across their gene bodies (Additional file 5: Figure S3). Given that DNA methylation can regulate gene transcription through changes in enhancer regions we examined the correlation between age-DMRs mapped to enhancer regions (determined by H3K27ac ChIP data from cortex) and transcriptional changes of their nearby genes. A significant negative correlation was observed between age-DMRs in enhancer regions and age-DEGs in both males (*r* = − 0.21, *p* = 0.018) and females (*r* = − 0.25, *p* = 0.04) (Fig. 2e, h). Age-DMRs mapped to gene bodies or enhancers associated with genes that were not differentially expressed with aging resulted in significant, but very weak negative correlation (*r* < 0.1) in both males and females (Fig. 2d, e, g, h). Taken together, age-DMRs may explain a small portion of the transcriptional changes that occur with age, and generally this effect is observed in enhancers and gene bodies, but not promoters. These findings are in agreement with recent studies in the liver showing a limited inverse association between gene body methylation with aging and gene repression of genes involved in lipid metabolism and growth hormone signaling [33]. Additionally, DNA methylation changes poorly correspond with transcriptional changes in the CNS during neuronal maturation [41] or following induction of methylation in culture [61]. Therefore, while the canonical regulation of gene transcription by DNA methylation is likely to explain a portion of the age-associated differential gene expression, age-related differential methylation may potentially serve a more complex role in transcriptional regulation than simply induction and suppression of steady-state gene expression.

### Age-related gene expression changes are associated with methylation profiles in early life

**Fig. 3:**
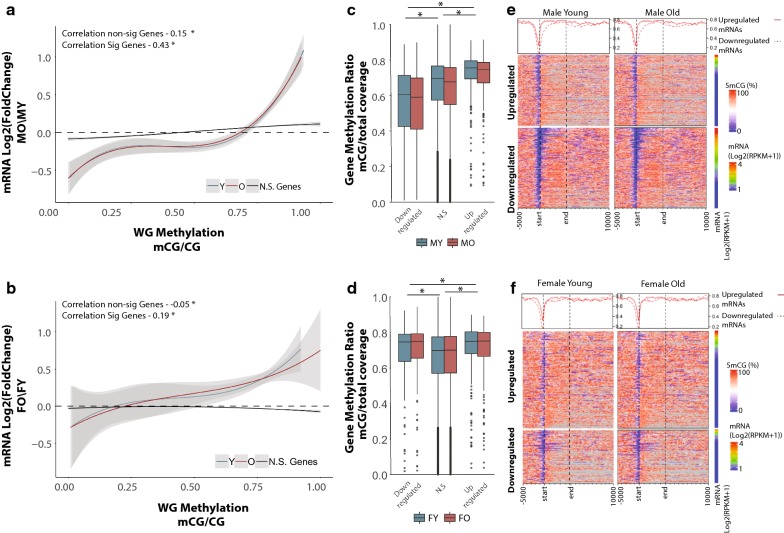
Age-related differentially expressed genes are positively associated with gene body methylation. Genes downregulated with aging have lower gene body methylation at young age (Y, blue regression line) in both males (**a**) and females (**b**) compared to genes upregulated with aging. This relationship is maintained in old age (O, red regression line). Curve corresponds to polynomial regression curve across significant (red and blue) and non-significant (N.S., black) differentially expressed genes, 95% confidence intervals are shaded by the grey area. Gene body methylation was calculated as methylation of all cytosines between the transcription start site and transcription end site of a given gene. Box plot of whole gene methylation grouped by genes upregulated, non-differentially expressed, and downregulated genes in males (**c**) and females (**d**) *p < 0.001 (Kruskal–Wallis Test). Heatmaps illustrating the per-gene gene body methylation patterns of genes upregulated and downregulated with aging in young and old, male (**e**) and female (**f**) animals

DNA methylation can play multiple roles in regulating gene transcription by altering protein binding occupancy [62], regulation of alternative splicing [63–67], and through interactions with histone marks [11, 68]. To examine relationships between DNA methylation patterns and gene expression with aging and gene body methylation levels (mean methylation from TSS to TES) (Fig. 3a, b) in early and late life were examined. Intriguingly, genes differentially expressed with aging show a moderate positive association between age-related differential mRNA expression and gene body methylation levels at both young and old age (Fig. 3a, b). Genes whose expression does not change with aging do not show a consistent positive association as observed for differentially expressed genes. That is, genes that were downregulated with aging have lower gene body methylation levels in early life, and remained lower to old age as compared to genes that were upregulated with aging (Fig. 3c, d). This relationship was consistent in both young and old animals and was not influenced by age-related changes in CpG methylation (Fig. 3c, d). This analysis was repeated for CH methylation to examine whether the relationship between early-life methylation and gene expression persists for non-CpGs. Unlike CpGs, CH methylation was comparable between upregulated genes and downregulated genes (Additional file 6: Figure S4A, B). The lack of interaction between CH methylation and changes in transcription may stem from the differences in functions between CpG and CH methylation in transcription regulation. Although transcriptional changes with aging are predominately sex-specific, this association was evident in both males and females (Fig. 3), with males showing a stronger association as compared to females.

**Fig. 4:**
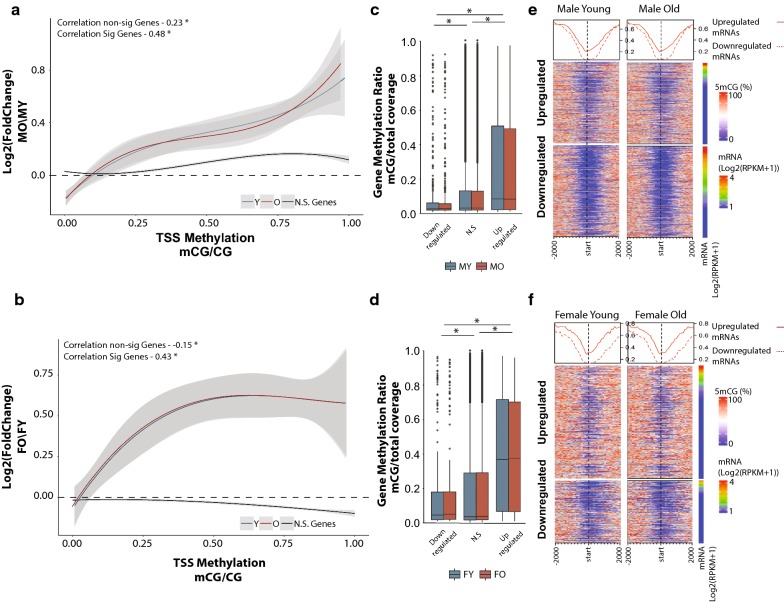
Age-related differentially expressed genes are positively associated with promoter methylation. Genes downregulated with aging have lower promoter methylation at young age (Y, blue) in both males (**a**) and females (**b**) compared to genes upregulated with aging. This relationship is maintained with aging (O, red). Curve corresponds to polynomial regression curve across significant (red and blue) and non-significant (N.S., black) differentially expressed genes, 95% confidence intervals are shaded by the grey area. Promoter is defined as ± 1 kb from transcription start site. Box plots of promoter methylation grouped by genes upregulated, non-differentially expressed, and downregulated genes in males (**c**) and females (**d**) *p < 0.001 (Kruskal–Wallis Test). Heatmaps illustrating promoter methylation patterns of genes upregulated and downregulated with aging in young and old in male (**e**) and female (**f**) animals

Qualitative assessment of the DNA methylation landscape of up- and downregulated genes with aging revealed that the main difference between up- and downregulated genes occurs primarily around the transcription start site (Fig. 3e, f). Therefore, we repeated the analysis focusing on promoter methylation defined as ± 1 kb of the TSS. The positive association between differentially expressed genes and baseline DNA methylation was recapitulated when examining only the promoter region (Fig. 4a, b), and was comparable in both sexes (Fig. 4c–f, Additional file 6: Figure S4C, D). Genes that do not change in expression with aging showed a weaker association that was not consistent between males and females (Fig. 4a, b). The correlation between promoter methylation levels and gene expression changes was greater compared to observed with gene body methylation and was independent of apparent age changes in methylation. Our observation reveals a relationship between age-related gene expression changes and DNA methylation that depends on the methylation patterns established early in life rather than differential methylation with aging. To determine whether the positive association between DNA methylation patterns and transcriptional changes with aging is observed in other tissues, we performed our analysis using paired WGBS and RNA-sequencing in the liver [33] (data obtained from GEO:GSE92486). A positive relationship between fold change and gene body methylation was observed with the liver data similar to that observed in the hippocampus (Additional file 7: Figure S5). The lack of whole-genome bisulfite sequencing data with aging in other tissues prevents further extension and validation of relationship at this time.

### Association of methylation patterns with transcriptional changes with aging is not random

**Fig. 5:**
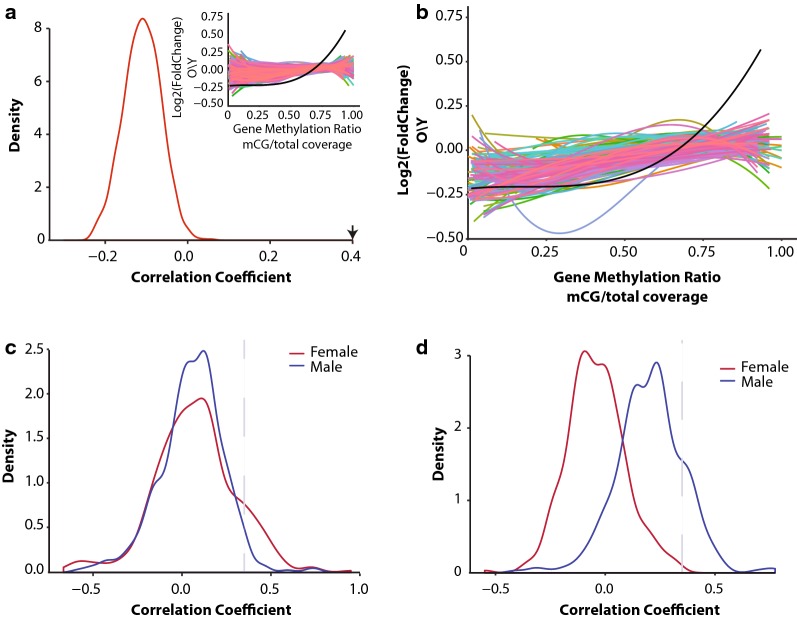
The association between differential expression and DNA methylation patterns in young animals is not random. **a** Distribution of the correlation coefficients generated by correlating log2 fold mRNA change with gene body methylation of 500 randomly sampled genes (*N* = 10,000). Arrow indicates the location of the correlation coefficient of gene body methylation and differentially expressed genes in males. Snippet showing the polynomial regression curves of randomly selected gene sets compared to that observed in males (black regression line). **b** Correlation between age-related differential gene expression and gene body methylation of Reactome pathways gene sets (only pathways with > 50 genes are included). Regression curve through all differentially expressed genes with aging and gene body methylation in males is shown in black. Distributions of the correlation coefficients generated by correlating log2 fold mRNA change with promoter (**c**) or gene body methylation (**d**) for each Reactome pathway gene set

Differentially expressed genes with aging appear to have a different DNA methylation profile compared to genes that are stably expressed across the lifespan (Figs. 3, 4). To determine whether this observation is unique to genes that are differentially regulated with aging, we used a random sampling approach to correlate gene body DNA methylation values with their corresponding mRNA fold change with aging. Randomly sampled sets of 500 genes (*n* = 10,000) showed weak correlation (*r* < 0.1) similar to that of genes not differentially expressed with aging and much less compared to that observed for genes differentially expressed with aging (*r* > 0.4) (Fig. 5a).

Next we asked whether genes sets that belong to the same pathway present a similar positive association. Pathways were extracted from the Reactome pathway database [69], and used as gene sets for correlation between methylation levels at young age and mRNA fold change with aging. After filtering pathways containing < 50 genes, 368 pathways remained for the analysis (Fig. 5b). Out of all the pathways analyzed, 35 pathways showed a correlation coefficient that met or exceeded the correlation coefficient of *r* > 0.4 (Fig. 5c) observed between promoter methylation and genes differentially expressed with aging. For gene body methylation 32 pathways met or exceeded the correlation coefficient cutoff (Fig. 5d) and were observed only in males. Pathways that showed the highest correlation between DNA methylation patterns and transcriptional change with age were pathways previously shown to be involved with aging included inflammatory pathways (transcriptional regulation by RUNX1, MHC II signaling, interferon signaling), oxidative stress, proteolysis, cell senescence, epigenetic regulation, and estrogen signaling (Additional file 8: Table S3, Additional file 9: Table S4).

**Fig. 6:**
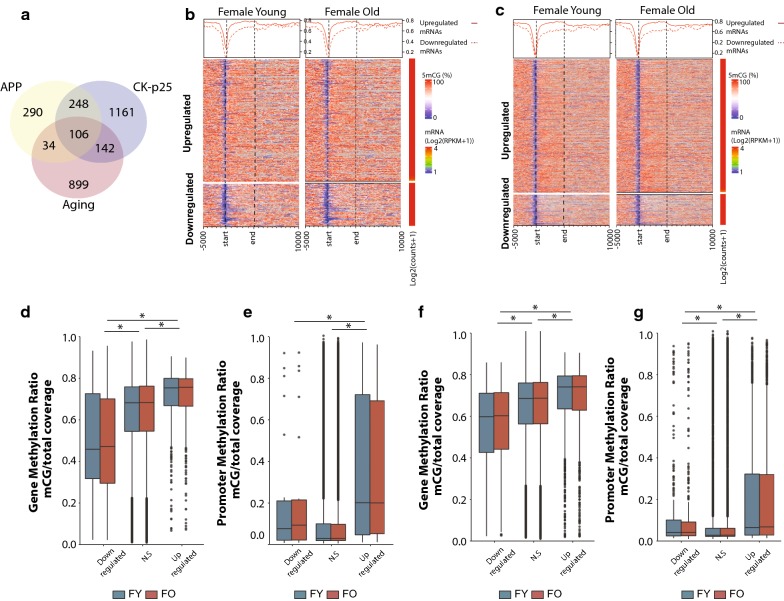
DNA methylation patterns in hippocampus of young and old animals are associated with genes differentially regulated in models of neurodegeneration. **a** Venn-diagram representing the overlap between genes differentially expressed in two models of neurodegeneration (APP and CK-p25) and genes differentially regulated with aging (males and females combined). Heatmaps illustrating the per-gene gene body methylation patterns of young and old animals (females only) in genes upregulated and downregulated in two models of neurodegeneration (**b** APP, **c** CK-p25). Box plots of gene body (**d**, **f**) and promoter (**e**, **g**) methylation grouped by genes upregulated, unchanged or downregulated in APP (**d**, **e**) and CK-p25 (**f**, **g**)

A central geroscience concept is that age-related changes intersect with those involved with disease pathogenesis, including Alzheimer’s disease [18, 70]. Therefore, we hypothesized that a positive correlation between transcriptional changes with neurodegeneration and DNA methylation profiles would be observed. To identify genes altered following neurodegeneration in the hippocampus, we used published RNA-sequencing data from two models of AD (APP and Ck-p25) and examined whether gene body and promoter DNA methylation levels in young and old animals are associated with differential gene expression observed in a neurodegenerative disease model. A significant number of genes were unique to each of the models; however, significant overlap was observed between both AD models and with genes altered with aging (APP:Aging *χ*^2^ *p* < 2.2 × 10^−16^; CK-p25:Aging *χ*^2^ *p* < 2.0 × 10^−14^; APP:CK-p25 *χ*^2^ *p* < 2.2 × 10^−16^) (Fig. 6a). As observed with genes differentially regulated with aging, upregulated genes with both APP and CK-p25 had significantly higher mean methylation in early life compared to downregulated genes (Fig. 6b, c). This was observed for gene body (Fig. 6d, f) and promoter methylation (Fig. 6e, g) as well. Differences in methylation in these models were not examined, therefore a potential difference in methylation due to AD pathology as a driving mechanism of differential gene regulation cannot be excluded; however, our findings suggest that genes differentially regulated with neurodegeneration may be more susceptible to change due to their methylation profile in a manner similar to that observed for genes differentially expressed with aging.

### DNA methylation-based prediction of differential expression with aging

**Fig. 7:**
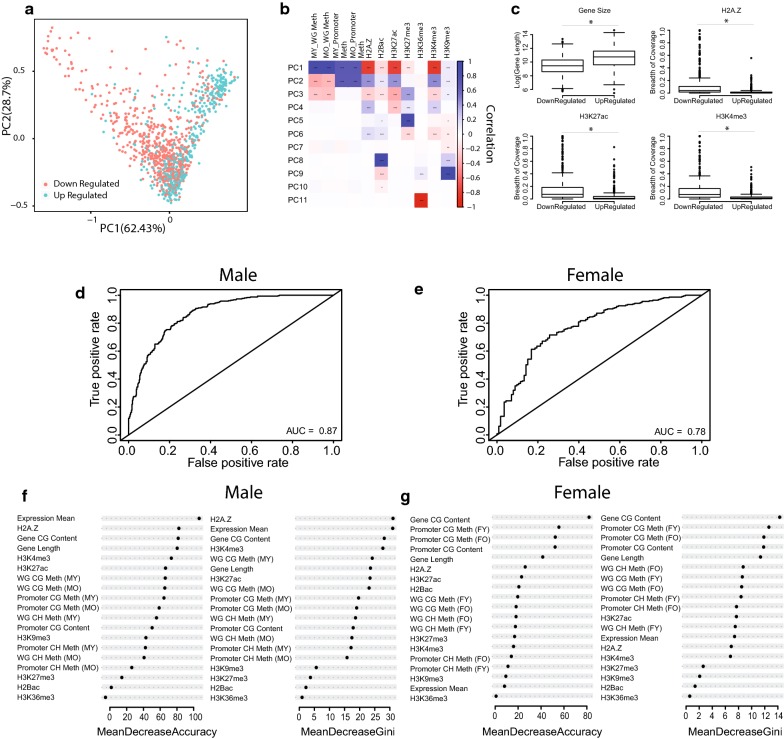
Direction of change of age-related differentially expressed genes can be predicated based on epigenetic marks in young age. Principle component analysis of epigenetic profiles of upregulated and downregulated genes with aging in the hippocampus (**a**). Correlation matrix representing the correlations between each principle component with epigenetic marks (**b**). Box plots comparing highly correlated epigenetic marks with the first principle component in upregulated and downregulated genes with aging (**c**). Area under the curve of the receive operating characteristic (ROC) curve showing the classification accuracy of differentially expressed to upregulated and downregulated genes for Random Forest model in males (**d**) and females (**e**). Feature importance of epigenetic marks for classification accuracy (mean decrease accuracy and mean decrease gini) in males (**f**) and females (**g**)

Given the distinction in early-life methylation patterns among age-related differentially expressed genes, we investigated the early-life patterns of other epigenetic marks known to interact with DNA methylation in genes that are up and downregulated with aging. Using publicly available data sets of histone marks maps generated from the young mouse hippocampus and cortex (H2Bac, H3K27ac, H3K27me3, H3K36me3, H3K4me3, H3K9me3, and H2A.Z), we profiled each age-related differentially expressed gene’s epigenetic landscape using DNA methylation data and the gene’s calculated histone breadth of coverage. A principal component analysis (PCA) based on genes’ epigenetic profiles revealed a separation between upregulated genes and downregulated genes. Combined PC1 and PC2 explained 90% of the variance between upregulated and downregulated genes (Fig. 7a). Correlation of the first component eigenvectors with the original epigenetic variables showed strong positive correlation with DNA methylation and negative correlation with active transcription marks such as H3K27ac, an active enhancer mark and H3K4me3, an active promoter mark (Fig. 7b). This suggests that at baseline (young age), genes that undergo expression changes with aging are under different epigenetic regulation during earlier time-points. Interestingly, the second principle component (variance explained 28.7%) shown the opposite correlation as the first components and was negatively correlated with gene body methylation and active transcription marks (Fig. 7b). Together this shows that genes differentially expressed with aging have different epigenetic patterns, starting in early life. This early-life epigenetic landscape may alter these genes’ responsivity to aging. As expected, not all genes differed according to their epigenetic profile. A subset of genes showed a similar epigenetic profile regardless of their expression trajectory.

Next, we set to investigate the associations between different epigenetic marks in age-related differentially expressed genes. Genes were separated by up and downregulation with aging and the interactions between the different epigenetic marks were investigated. While the baseline epigenetic profile of genes appear to differ between up and downregulated genes (Figs. 3, 4, 7a, b), the interactions between these epigenetic marks remain consistent between up and downregulated genes. Promoter and gene body methylation were positively correlated with one another in both gene groups, and as expected were negatively correlated with active enhancer and promoter marks, H3K27ac and H3K4me3 (Additional file 10: Figure S6A, B). While the interactions between epigenetic marks did not change between differentially expressed genes with aging, similar to DNA methylation levels, the baseline levels of different histone marks were different between up and downregulated genes. Genes that were downregulated with aging show higher breadth of coverage of active transcription marks compared to upregulated genes (Fig. 7c). This is consistent with the lower promoter methylation levels observed in these genes. Interestingly, the gene size of up and downregulated genes was also different between up- and down-age-related differentially expressed genes with upregulated genes significantly longer than downregulated genes (Fig. 7c). Together, these findings further demonstrate that altered epigenetic patterns may contribute to the trajectory of change of genes changing with aging.

To strengthen the potential link between differences in epigenetic landscape in young age and differential expression observed late in life we used random forest (RF) modeling to find whether early-life epigenetic patterns can predict gene expression changes with aging. The RF models were trained to predict the direction of transcriptional change with age (upregulated or downregulated) based on methylation data, gene size, relative expression in young age expressed by RPKM, and the epigenetic marks annotated in the hippocampus and cortex obtained from publicly available data sets (see methods).

The trained RF model was able to correctly classify transcriptional changes with high accuracy in both males (87%) and females (78%) (Fig. 7d, e). RF performance decreased slightly when trained based on DNA methylation means and RPKM alone, but still performed significantly better than random in both males (78%) and females (71%) (Additional file 10: Figure S6C, D). Evaluation of feature importance to each of the RF models revealed that DNA methylation and gene size are highly important for predicting gene expression in both sexes. In males, gene size, H2A.Z marks, H3K4me3, H3K27ac, and DNA methylation averages of both whole gene and promoters (Fig. 7f) contributed the most to predictive accuracy. In females, high importance features for model prediction included mean expression, DNA methylation levels and gene size (Fig. 7g). Feature importance measures of histone breadth of coverage were much lower in females compared to males. This is likely due to well-documented sex-differences in histone landscape observed in both mice and humans [71], which were not accounted for in the current analysis as most histone data available for hippocampus obtained for the analysis were collected from male animals.

It should be noted that these different epigenetic marks are not independent of each other as DNA methylation is closely associated with both H3K4me3, an active promoter mark [72], and H3K27ac, an enhancer mark [73]. Regions of H3K4me3 and H3K27ac often act coordinately with DNA methylation during gene transcription regulation [74]. Local depletion of DNA methylation is a hallmark of H3K4me3 and H3K27ac [56], and thus these marks are considered to be regulated by DNA methylation. Gene size was a significant contributor to the accuracy of the models (Fig. 7c, d), the relationship between gene length and DNA methylation is still not completely understood; however, transcription of long genes may be partially regulated by DNA methylation. For example, in the CNS transcriptional regulation of long genes is mediated through the DNA methylation binding protein MeCP2 [75]. The results presented here are in agreement with those of Benayoun et al. [76] which examined some of these marks but not DNA methylation in the cerebellum and olfactory bulb. Taken together, these results put forward the concept that epigenetic regulation at a young age may direct transcriptional change with aging.

## Discussion

These studies reveal, by analyzing the methylation and transcriptional profiles in the hippocampus of young and old animals, evidence for a potentially novel role for DNA methylation in regulating transcriptional changes with age that is independent of age-related changes to the methylome. These data demonstrate a propensity for genes to be upregulated or downregulated in expression with aging based on their methylation profiles established early in life. Additionally, differences in methylation with age are enriched in exonic and intronic regions, and showed a weak inverse correlation with differences in gene expression. The functional role of gene body methylation is not yet well defined but is associated with transcriptional elongation [77], splicing [64, 65, 67, 78], regulation of alternative promoters [79], and modulation of expression levels through interaction with methyl-binding proteins such as MeCP2 [80, 81]. In the CNS, in contrast to other tissues, gene body methylation is inversely correlated with expression levels [1, 82], a relationship observed here. The diverse functional roles of gene body methylation create a challenge in interpreting the association between gene body age-DMRs and the altered transcriptional profile with aging. Nevertheless, age-related differential methylation within genes is common to various tissues; therefore, improved knowledge on how gene body methylation regulates expression is required to understand the potential functions age-DMRs play in regulation of the aging transcriptome. Together these findings emphasize the importance of gene body methylation, in addition to promoter regions, as a gene expression regulatory mechanism.

### Association of promoter age-DMRs with age-DEGs is limited

The association between DNA methylation and gene expression is often derived from the inverse correlation between mRNA expression and DNA methylation in promoters under normal conditions [8]. While differences in promoter methylation in the hippocampus occur with aging, the genes associated with these promoters are generally not differentially expressed with age (Fig. 2). A potential explanation is that observed changes to the methylome with age are subtle and therefore insufficient to induce transcriptional differences, however, a weak correlation between gene expression changes and differential promoter methylation is also observed in studies of cancer and cellular differentiation [83, 84], which include disruption to- (cancer) or reprogramming of- (differentiation) the methylome. The limited correlation between age-related differential promoter methylation and gene expression changes does not preclude differential promoter methylation from altering expression of specific genes, but is insufficient to explain the majority of transcriptional changes observed with age in the hippocampus. It should also be noted that gene expression changes rapidly with stimuli and the expression levels here were collected to represent steady-state expression levels. As well, examination of specific call types or even single cells is needed as these data represent a mix of cell types present in the hippocampus.

### Enhancer age-DMRs are related to age-DEGs

Recent studies identified that altered DNA methylation patterns play a greater role in explaining transcriptional changes when they occur in distal regulatory regions, namely enhancers, compared to gene promoters [84]. Age-related differential methylation is enriched in enhancer marks in various tissues [37, 85–87], including in the hippocampus [26]. With aging, altered methylation in differentiating cells, specifically hypomethylation, was shown to be enriched in regions marked by H3K4me1 [88], a marker of active and poised enhancers [89], and is thought to activate gene expression. Consistent with these findings, we found enrichment of both hyper- and hypo-methylated age-DMRs in regions distal to gene promoters, specifically in annotated active and poised enhancers. These age-DMRs were inversely correlated with transcriptional differences with aging in both males and females.

Recent findings shed light on the interaction between the enhancer marks H3K27ac and H3K4me1 and DNA methylation and the functional role of this interaction on gene transcription regulation [90]. Enhancer activation can be both positively or negatively associated with DNA methylation depending on the regulatory nature of the enhancer and the developmental stage of the organism [55, 56, 91]. Enhancers containing transcription factor binding motifs tend to be inversely correlated with DNA methylation late in life, but not during cellular differentiation where DNA methylation increases in enhancers proximal to genes that involve cellular specification [74]. Methylation of super-enhancers is thought to contribute to the structural integrity of the genome at these regions [91, 92]. Although alterations in chromatin landscape with aging have been reported, few studies have mapped altered histone marks with age. H2A.Z, a histone variant needed for the acetylation of histone 3 lysine 27 [93], does change with aging in the hippocampus [94], and may be a contributing mechanism to enhancer mark changes with aging. Given these results we hypothesize that changes in methylation can potentially alter transcription through attenuation of enhancer strength rather than facilitating deposition of H3K27ac. Future studies will need to address this hypothesis by mapping the differences in enhancer landscape with age in both male and female and in different cell/tissue types.

A unique feature of genes that were differentially expressed with age was their association with DNA methylation patterns established in early life (i.e., methylation levels in young animals). Methylation levels of upregulated genes were higher than levels of downregulated genes in young animals, this difference persists in old animals and therefore was generally independent of age-related differential methylation. The association between methylation levels and expression was not observed for genes that were not altered with aging or randomly selected genes. Furthermore, gene expression changes with aging were generally different between males and females, yet a similar association was observed in both sexes. This finding supports the concept that, based on their epigenetic patterns established early in life, specific genes have a higher propensity to change with age than others and that their induction/reduction is dependent on the methylation status of the gene. Therefore, suppression or induction of genes with aging is likely to occur downstream of methylation by factors that interact with the methylome such as histone modifications or methyl-binding protein dynamics. An additional finding was that genes that changed with age and correlated with early-life methylation occur in specific gene sets that function in similar pathways. This is consistent with the notion that genes with similar functions are regulated in similar ways [95, 96].

Using the predictive capabilities of machine learning we were able to show that baseline gene expression and DNA methylation levels alone can classify whether differentially expressed genes will be downregulated or upregulated. When other epigenetic marks from the young/adult brain are added to the model, the classification accuracy of the model improves. This provides further support to the idea of early epigenetic programming as a determining factor of expression changes with age. A recent study [76] showed similar results by predicting age-related expression changes based on chromatin marks. The authors found that changes in the enhancer mark H3K27ac with age were among the highly important features for accurate classification. This indicates that age-related alterations to the epigenome contribute to transcriptional changes with age. Although changes in chromatin predict gene expression changes well, we were able to achieve similar predictive capabilities based on early-life DNA methylation alone, and in both males and females. Future studies combining both baseline epigenetic profiles and age-related alteration to histones are needed to improve the classification accuracy of these models, and perhaps help identify the interplay between mechanisms that underlie epigenetic regulation of transcriptional changes with aging.

Aging processes are thought to promote the development of age-related neurodegenerations like AD and PD [47]. In our study we find that the association between early-life methylation patterns and differential gene expression is also observed in genes that are dysregulated in mouse-models of AD. That is, genes that were upregulated in a model of neurodegenerative disease had higher gene body methylation at young age compared to those that were downregulated. Thus, it is plausible that DNA methylation patterns established at young age may facilitate transcriptional changes and more severe conditions in late life as well. Given that genetic differences are associated with age-related transcriptional differences [97], and increased longevity in supercentenarians [98, 99] it is entirely plausible that early-life epigenetic patterns could have similar impacts. This raises the question of what may cause epigenetic differences in early life that have late-life outcomes.

Early-life events such as differences in maternal care, nutrition, or adverse events can cause long-lasting alterations to the neuroepigenome [100–102]. Therefore, epigenomic programming during developmental stages and early adulthood may serve as a potential mechanism for altered late-life outcomes, including aging and susceptibility to disease. In addition, DNA methylation patterns are also altered with anti-aging therapies that have a beneficial effect on molecular and cellular aging hallmarks [26, 37]. These therapies, for example calorie restriction, have been shown to be potent in a short window early in life and following life-long treatment [103, 104]. A point for further investigation is how these anti-aging therapies can alter methylation patterns both early and late in life to prevent age-related transcriptional changes and promote a pro-longevity phenotype.

## Conclusions

Age-related differences in epigenetic marks are likely to contribute to transcriptional alterations, however, these epigenetic differences account for a small subset of the gene expression changes with aging and are dependent on the genomic location, e.g., promoter vs. regulatory region. It is noteworthy that our current knowledge of the exact location of regulatory marks is far from complete and is likely to vary between cell types, tissues, and sexes. It would be important to test predictive validity with improved and more complete data sets as well as refined locations of TSSs, alternative splice sites, and gene regulatory marks. Our current findings identify a potential new way in which DNA methylation can influence age-related transcriptional change. The early establishment of DNA methylation patterns of a gene appears to partially determine whether the gene will change with age and the directionality of the change. Interestingly, a recent manuscript identified a similar finding examining histone modifications in the cerebellum and olfactory bulb [76]. We also observed this association with aging in the liver and in Alzheimer’s disease models. Together, these findings indicate that the early-life epigenetic landscape of a gene may direct its gene expression trajectory with aging and age-related disease. These findings provide a potential mechanism for the developmental origins of disease concept [105].

## Materials and methods

### Animals and nucleic acid extraction

Male and female C57BL/6 mice were obtained from the NIA aging colony at 2 and 21 months of age. Mice were housed at the University of Oklahoma Health Sciences Center barrier animal facility and maintained under SPF conditions until 3 and 24 months of age. All experimental procedures were performed according to protocols approved by the OUHSC Institutional Animal Care and Use Committee. Mice were euthanized by decapitation and hippocampal tissue was dissected and snap-frozen until used for DNA and RNA extraction. DNA and RNA from young and old animals (*n* = 6/group) were isolated from hippocampal tissue using Zymo Duet DNA/RNA (Zymo research).

### Whole-genome bisulfite sequencing and DMR calling

Isolated genomic DNA from young and old animals (*n* = 3/group) was used for Whole-Genome Bisulfite Sequencing (WGBS). Bisulfite conversion was carried out using EZ DNA methylation Lighting (Zymo Research, Irvine, CA) and library construction used Swift Accel-NGS methyl-seq kit reagents (Swift Bioscience, Ann Arbor, MI) following manufacturer’s instructions. Library size was assessed by Tapestation (Agilent Technologies, Santa Clara, CA) and quantified by quantitative PCR (Kappa Biosystems, Wilmington, MA) prior to sequencing. BS-seq libraries were sequenced by 100 bp paired-end reads on the Illumina HiSeq-2500 (Illumina, San Diego, California, USA). Sequencing data will be made available upon manuscript submission.

Paired-end reads were trimmed using trimmomatic version 0.35 [106]. Reads were adapter-trimmed and filtered based on quality. Bases with a Q-score < 30 were removed from the 5′ and 3′ ends. Reads were quality-filtered using a sliding window approach (parameters were set to 5:30). Additionally, reads shorter < 25 bp post-trimming were removed. Trimmed PE reads were aligned to the mouse reference genome (GRCm38/mm10) with Bismark Bisulfite Mapper version 0.14.4 [107] using default settings. Methylation % and coverage of each CpG site were extracted with bismark methylation extractor. Mean coverage per sample was 5× (± 0.4 SD). For differentially methylated regions calling, sites with < 5× mean coverage per group were removed resulting based previous sequencing recommendations [108] in > 20 million CG sites analyzed (Additional file 11: Figure S7).

To determine differentially methylated regions (age-DMRs), the genome was binned into consecutive, non-overlapping 500 bp windows. Samples within each group were combined to achieve higher coverage per site, and windows with < 10 CpG sites were omitted from the analysis (Additional file 11: Figure S7). The number of CpGs per widow was determined based on approximation of CpG density, Statistical significance of differential methylation was determined using Fisher’s exact test followed by false-discovery multiple testing correction. Differentially methylated regions were considered statistically different if FDR-adjusted *p* value < 0.05.

### RNA sequencing and differential gene expression analysis

RNA integrity was quantified by TapeStation (Agilent Technologies, Frankfurt, Germany) and samples had RNA integrity numbers > 8. RNA-sequencing libraries were prepared using Illumina’s TruSeq RNA-seq library prep with a rRNA depletion step according to manufacturer’s instructions. Libraries were sequenced with 150 bp paired-end (PE) reads on the Illumina HiSeq 4000 platform (Illumina, San Diego, California, USA) (*n* = 6/group). Sequence quality control was performed with fastQC. Following QC step PE reads were trimmed similarly to the WGBS sequences using trimmomatic.

Following QC and trimming, reads were aligned to the mouse (mm10) reference genome using STAR [109]. For alignment, the genome was prepared based on GENCODE M15 release. STAR Alignment parameters were set to: outFilterScoreMin 2, outFilterMultimapNmax 5, outFilterMismatchNmax 10, outFilterMatchNmin 20, outSJfilterReads Unique, outSJfilterOverhangMin 25 10 10 10, alignSJoverhangMin 2, alignSJDBoverhangMin 2, chimSegmentMin 25. Reads per gene were counted in R using the ‘summarizeOverlap’ function in the GenomicAlignments package. Raw reads were normalized using DESeq 2 R package [110] and transformed using variance stabilized transformation. Differential expression between all groups was assessed using multiple linear regression (R package ‘glm’) using read counts as the dependent variable and age (young and old) and sex (male and female) as the independent variables. Genes with significant age main effect (*p* < 0.05) were then carried on for pair-wise comparisons using Conover post-hoc test followed by false discovery rate adjustment using ‘fdr’ as implemented in the R package ‘lsmeans’.

### Enrichment analysis

For pathway enrichment age-DMRs were annotated using ChIPseeker [111], and enrichment analysis was performed using the R package ‘ReactomePA’ [112]. To determine over- and under-representation of DMRs in genomic features, annotated introns, exons, and CpG islands were obtained from UCSC Genome Browser. Promoters were defined as ± 1 kb from the transcription start site. CpG shores were defined as 2 kb upstream and downstream of the annotated CpG island boarders and CpG shelves were defined as 2 kb upstream and downstream from shores. Of the 18,000 genes identified to be expressed in our set, > 94% had a DMR mapped to their gene body, and > 85% had a DMR mapped to their promoter (Additional file 11: Figure S7). Gene-regulatory regions in the mouse brain were extracted from Ensemble open database [113]. DMRs were mapped to genomic features using ‘bedtools’ [114]. Statistical significance of over- or under-representation of DMRs in genomic features was determined using hypergeometric test in R.

### Differential expression prediction

Differentially expressed mRNAs with aging were classified based on the directionality of change (upregulated or downregulated) and divided into a training set and a validation set by randomly subsetting 70% of the genes to the training set, the remaining genes were used for model validation. Prediction of gene change directionality with aging was performed separately for male and females. Random forest (RF) was used for prediction, and all analysis and cross-validation was performed in R using the ‘randomforest’ package. The RF model was trained based on selected epigenetic features including mean gene DNA methylation in young and old, mean promoter (± 1 kb of TSS) methylation in young and old, gene size, base expression at 3 months, and breadth of coverage of the following histone marks: H2A.Z from young and old animals, H3K27ac, H3K36me3, H3K4me3, H3K27me3, H2Bac, and H3K9me3. Breadth of coverage was calculated by the breadth sum of all peaks in a gene/gene length.

### Public data acquisition

Paired methylation and differential expression data for liver were obtained from GEO:GSE92486 [33]. Differential genes expression for age-related neurodegenerative disease APPswe/PS1ΔE9 (APP) and Ck-P25 models were obtained from GEO:GSE93678 [115] and GEO:GSE65159 [116]. Only WT control and experimental groups were used. ChIP-sequencing data of hippocampal histone marks were obtained from GEO:GSE85873 (H3K4me3 and H3K27me3) [117], GEO:GSE103358 (H2Bac), and GEO:GSE100039 (H2A.Z) [94]. Cortex epigenetic marks including H3K27ac, H3K36me3, and H3K9me3 were obtained from GEO: GSE103214 [118]. Peak calling was determined with MACS2 [119].

## Supplementary information

**Additional file 1: Figure S1.** Distribution of gene body methylation using RRBS and WGBS. A comparison of the distribution of gene body methylation across all genes in the liver measured by reduced representation bisulfite sequencing (RRBS) and whole-genome bisulfite sequencing (WGBS) obtained from GEO:GSE92486. RRBS covers methylation over portions of 23,000 genes as compared to near complete coverage of 29,000 genes by WGBS. The gene body methylation profiles obtained by RRBS do not represent the gene body methylation values observed by WGBS, likely in part due to the preference of RRBS for regions of high CG density which often have low levels of methylation (e.g., CpG Islands).

**Additional file 2: Figure S2.** Global DNA methylation levels measures using WGBS. A) Box plots of whole-genome methylation in young and old males and females. Box plots showing the methylation levels of cytosines mapped to all transposable element regions (B) or to specific transposable element families. Long interspersed nuclear repeats, LINEs (C), small interspersed nuclear repeats, SINEs (D), DNA transposons (E), and long terminal repeats, LTRs (F), *n* = 3/group.

**Additional file 3: Table S1.** List of Reactome pathways enriched with age-related DMRs.

**Additional file 4: Table S2.** List of Reactome pathways enriched with age-related DMRs separated by hypermethylation and hypomethylation.

**Additional file 5: Figure S3.** Baseline gene body methylation is not different in age-related differentially expressed genes from those who do not change with age. A-F) line plots representing the average methylation across all genes that were not significantly differentially expressed with aging (A, D), downregulated with aging (B, E), and upregulated with aging (C,F) in females (A-C) and males (D-F). Black line represents young animals, red line represents old animals. Grey shading represents the 95% confidence intervals.

**Additional file 6: Figure S4.** Positive association between methylation and gene expression is limited for CH methylation. Box plots of whole gene (A, B) or promoter (C, D) CH methylation in females (A, C) and males (B, D) grouped by genes that are upregulated, downregulated, or unchanged with aging in the respective group.

**Additional file 7: Figure S5.** Age-related differentially expressed genes are positively associated with gene body methylation in the Liver A) Genes downregulated with aging have lower gene body methylation at young age (blue regression line) compared to genes upregulated with aging in the liver. This relationship is maintained with aging (red regression line). Curve corresponds to the polynomial regression curve across significant (red and blue) and non-significant (black) differentially expressed genes. B) Box plot of whole gene methylation grouped by genes upregulated, non-differentially expressed, and downregulated genes in the liver. *p < 0.001 (Kruskal–Wallis Test).

**Additional file 8: Table S3.** Reactome pathways presenting high correlation between age-related expression fold change and early life gene body methylation patterns.

**Additional file 9: Table S4.** Reactome pathways presenting high correlation between age-related expression fold change and early life gene promoter methylation patterns.

**Additional file 10: Figure S6.** Direction of change of age-related differentially expressed genes can be predicted based on DNA methylation profiles. Correlation matrices of different epigenetic features in downregulated genes with aging (A) and upregulated genes with aging (B). Area under the curve of the receive operating characteristics (ROC) curve showing the classification accuracy of age-related differentially expressed genes to upregulated and downregulated genes for Random Forest model in males (C) and females (D) trained based on baseline methylation and promoter and gene body DNA methylation. TRatio—gene ratio; wg—whole gene; tss—transcription start site; my—male young; mo—male old.

**Additional file 11: Figure S7.** Sequencing alignment and differentially methylated region calling summary statistics. A. Boxplots representing the mapping efficiency per group. B. Overall genomic sequencing coverage. C. Boxplots showing the number of CpGs covered. D. Boxplots representing the average CpG coverage per group. E. Line plot of the number of DMRs mapped to genes (black and red) and gene promoters (green and blue) passed filtering from RNA-sequencing. F. Line plot showing the number of CpG in regions passed filtering for differential methylation.

## Data Availability

Raw sequencing data are submitted to the Sequencing Read Archive (SRA) under bioProject number PRJNA523985.
