# Supplementary material for: Early-life DNA methylation profiles are indicative of age-related transcriptome changes
Source: Epigenetics Chromatin. 2019 Oct 8;12:58. doi: 10.1186/s13072-019-0306-5 (PMC6781367; doi:10.1186/s13072-019-0306-5)
Supplement: Supplementary file 1 — Additional file 1: Figure S1. Distribution of gene body methylation using RRBS and WGBS. A comparison of the distribution of gene body methylation across all genes in the liver measured by reduced representation bisulfite sequencing (RRBS) and whole-genome bisulfite sequencing (WGBS) obtained from GEO:GSE92486. RRBS covers methylation over portions of 23,000 genes as compared to near complete coverage of 29,000 genes by WGBS. The gene body methylation profiles obtained by RRBS do not represent the gene body methylation values observed by WGBS, likely in part due to the preference of RRBS for regions of high CG density which often have low levels of methylation (e.g., CpG Islands). [file 13072_2019_306_MOESM1_ESM.pdf]

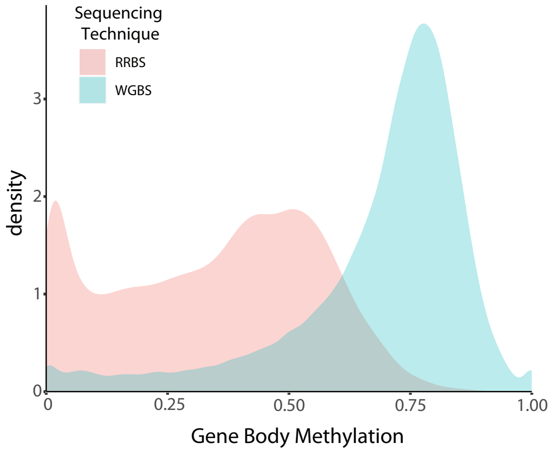

Supplemental Figure 1. Distribution of gene body methylation using RRBS and WGBS. A comparison of the distribution of gene body methylation across all genes in the liver measured by reduced representation bisulfite sequencing (RRBS) and whole genome bisulfite sequencing (WGBS) obtained from GEO:GSE92486. RRBS covers methylation over portions of 23000 genes as compared to near complete coverage of 29000 genes by WGBS. The gene body methylation profiles obtained by RRBS do not represent the gene body methylation values observed by WGBS, likely in part due to the preference of RRBS for regions of high CG density which often have low levels of methylation (e.g. CpG Islands).
