# Supplementary material for: Early-life DNA methylation profiles are indicative of age-related transcriptome changes
Source: Epigenetics Chromatin. 2019 Oct 8;12:58. doi: 10.1186/s13072-019-0306-5 (PMC6781367; doi:10.1186/s13072-019-0306-5)
Supplement: Supplementary file 2 — Additional file 2: Figure S2. Global DNA methylation levels measures using WGBS. A) Box plots of whole-genome methylation in young and old males and females. Box plots showing the methylation levels of cytosines mapped to all transposable element regions (B) or to specific transposable element families. Long interspersed nuclear repeats, LINEs (C), small interspersed nuclear repeats, SINEs (D), DNA transposons (E), and long terminal repeats, LTRs (F), n = 3/group. [file 13072_2019_306_MOESM2_ESM.pdf]

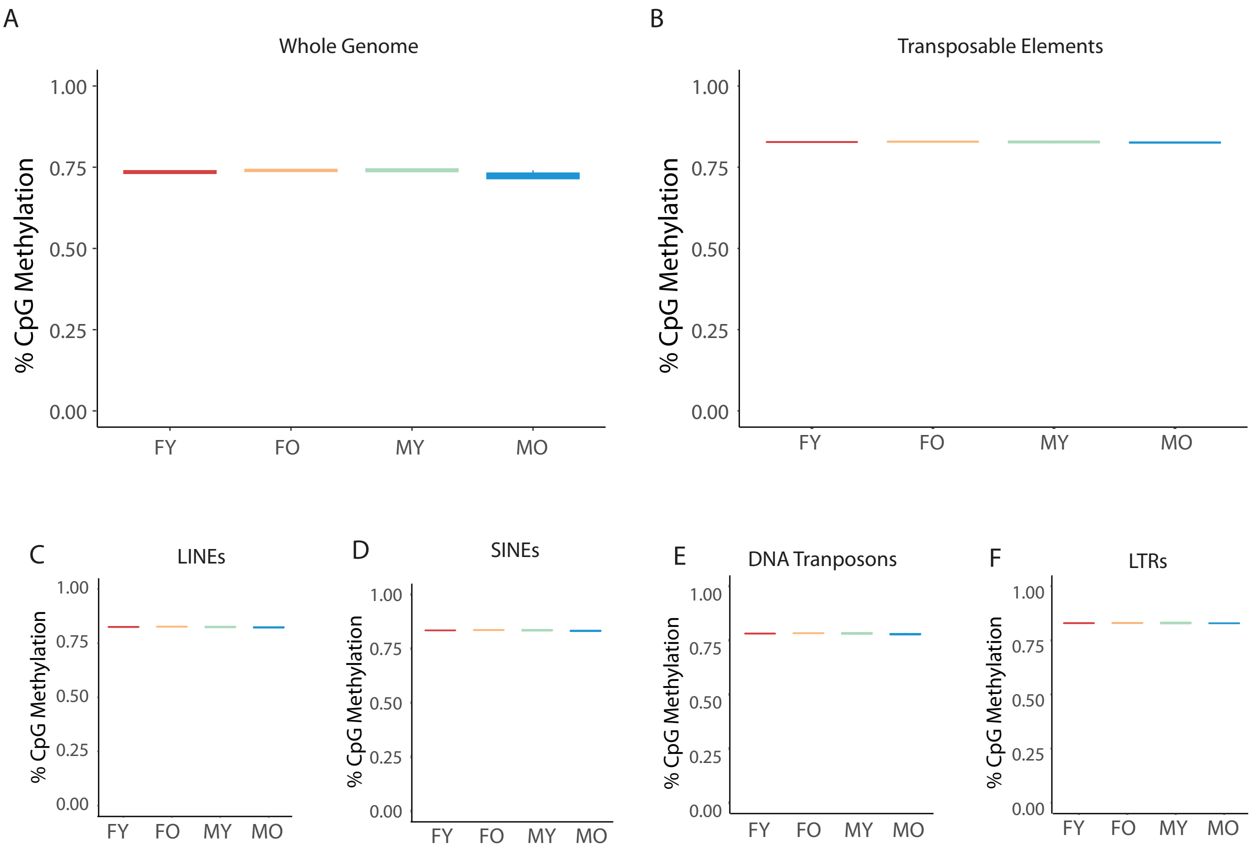

Supplemental Figure 2. Global DNA methylation levels measures using WGBS. A) Box plots of whole genome methylation in young and old males and females. Box plots showing the methylation levels of cytosines mapped to all transposable element regions (B) or to specific transposable element families. Long interspersed nuclear repeats, LINEs (C), small interspersed nuclear repeats, SINEs (D), DNA transposons (E), and long terminal repeats, LTRs (F), n=3/group.
