# Supplementary material for: Early-life DNA methylation profiles are indicative of age-related transcriptome changes
Source: Epigenetics Chromatin. 2019 Oct 8;12:58. doi: 10.1186/s13072-019-0306-5 (PMC6781367; doi:10.1186/s13072-019-0306-5)
Supplement: Supplementary file 5 — Additional file 5: Figure S3. Baseline gene body methylation is not different in age-related differentially expressed genes from those who do not change with age. A-F) line plots representing the average methylation across all genes that were not significantly differentially expressed with aging (A, D), downregulated with aging (B, E), and upregulated with aging (C,F) in females (A-C) and males (D-F). Black line represents young animals, red line represents old animals. Grey shading represents the 95% confidence intervals. [file 13072_2019_306_MOESM5_ESM.pdf]

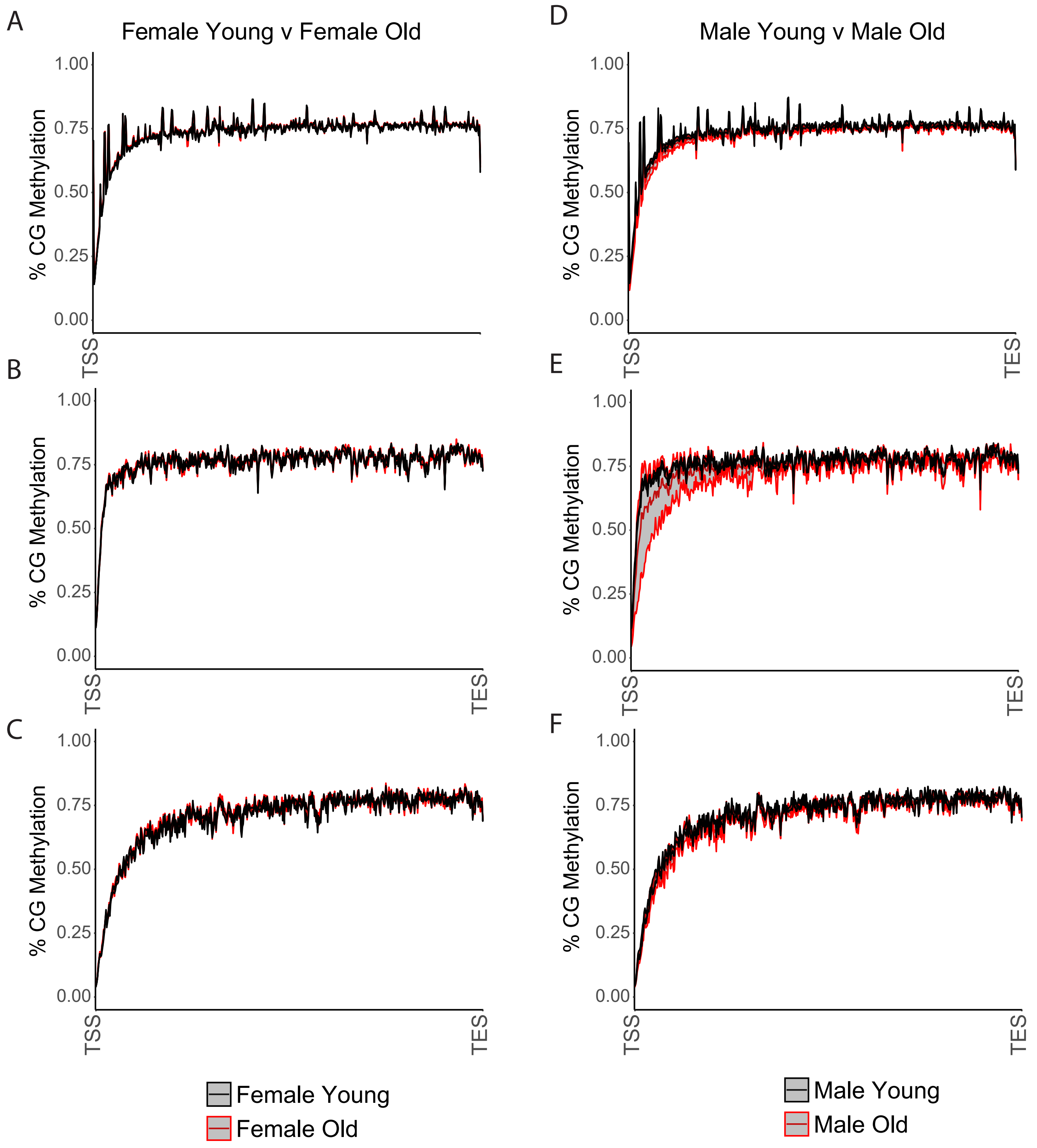

Supplemental Figure 3. Baseline gene body methylation is not different in age-related differentially expressed genes from those the do not change with age. A-F) Line plots representing the average methylation across all genes that were not significantly differentially expressed with aging (A,D), down-regulated with aging (B,E), and upregulated with aging (C,F) in females (A-C) and males (D-F). Black line represent young animals, red line represent old animals. Grey shading represent the 95% confidence intervals.
