# Supplementary material for: Early-life DNA methylation profiles are indicative of age-related transcriptome changes
Source: Epigenetics Chromatin. 2019 Oct 8;12:58. doi: 10.1186/s13072-019-0306-5 (PMC6781367; doi:10.1186/s13072-019-0306-5)
Supplement: Supplementary file 6 — Additional file 6: Figure S4. Positive association between methylation and gene expression is limited for CH methylation. Box plots of whole gene (A, B) or promoter (C, D) CH methylation in females (A, C) and males (B, D) grouped by genes that are upregulated, downregulated, or unchanged with aging in the respective group. [file 13072_2019_306_MOESM6_ESM.pdf]

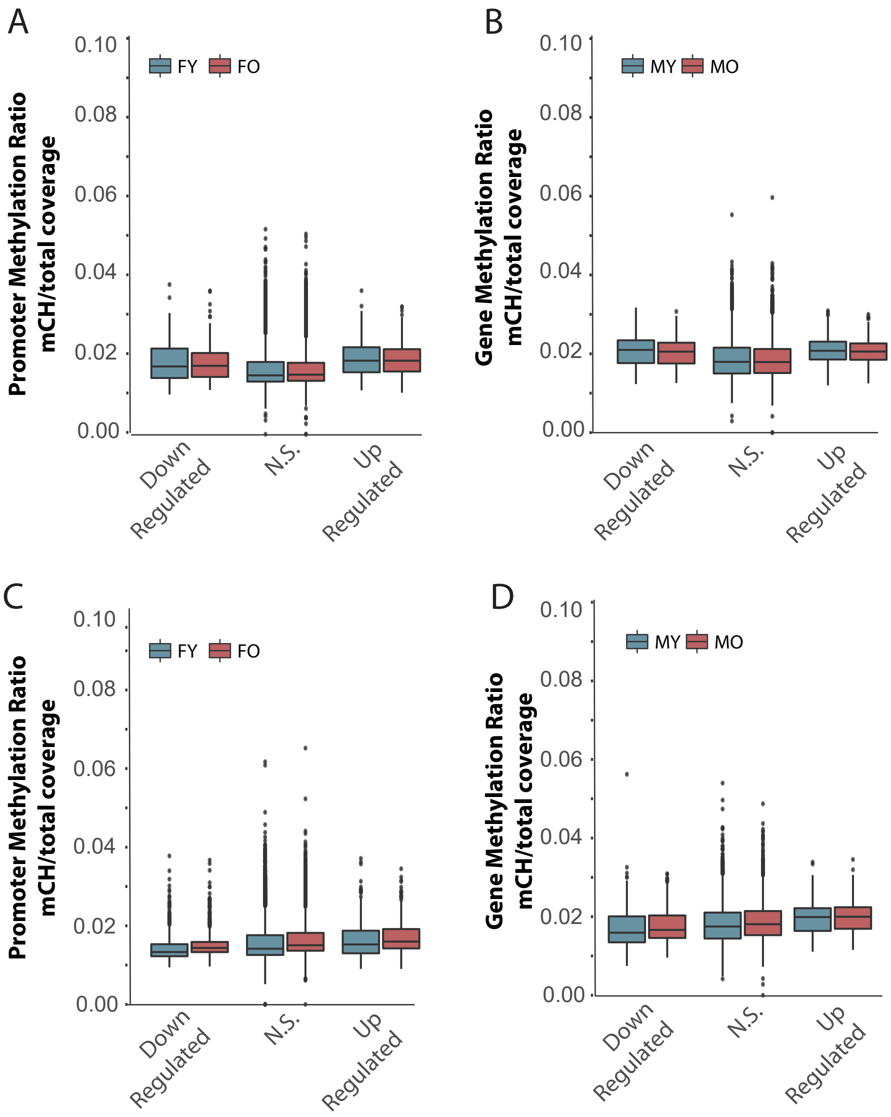

Supplemental Figure 4. Positive association between methylation and gene expression is limited for CH methylation. Box plots of whole gene (A,B) or promoter (C,D) CH methylation in females (A,C) and Males (B,D) grouped by genes that are up-regulated, down-regulated, or unchanged with aging in the respective group.
