# Supplementary material for: Early-life DNA methylation profiles are indicative of age-related transcriptome changes
Source: Epigenetics Chromatin. 2019 Oct 8;12:58. doi: 10.1186/s13072-019-0306-5 (PMC6781367; doi:10.1186/s13072-019-0306-5)
Supplement: Supplementary file 7 — Additional file 7: Figure S5. Age-related differentially expressed genes are positively associated with gene body methylation in the Liver A) Genes downregulated with aging have lower gene body methylation at young age (blue regression line) compared to genes upregulated with aging in the liver. This relationship is maintained with aging (red regression line). Curve corresponds to the polynomial regression curve across significant (red and blue) and non-significant (black) differentially expressed genes. B) Box plot of whole gene methylation grouped by genes upregulated, non-differentially expressed, and downregulated genes in the liver. *p < 0.001 (Kruskal–Wallis Test). [file 13072_2019_306_MOESM7_ESM.pdf]

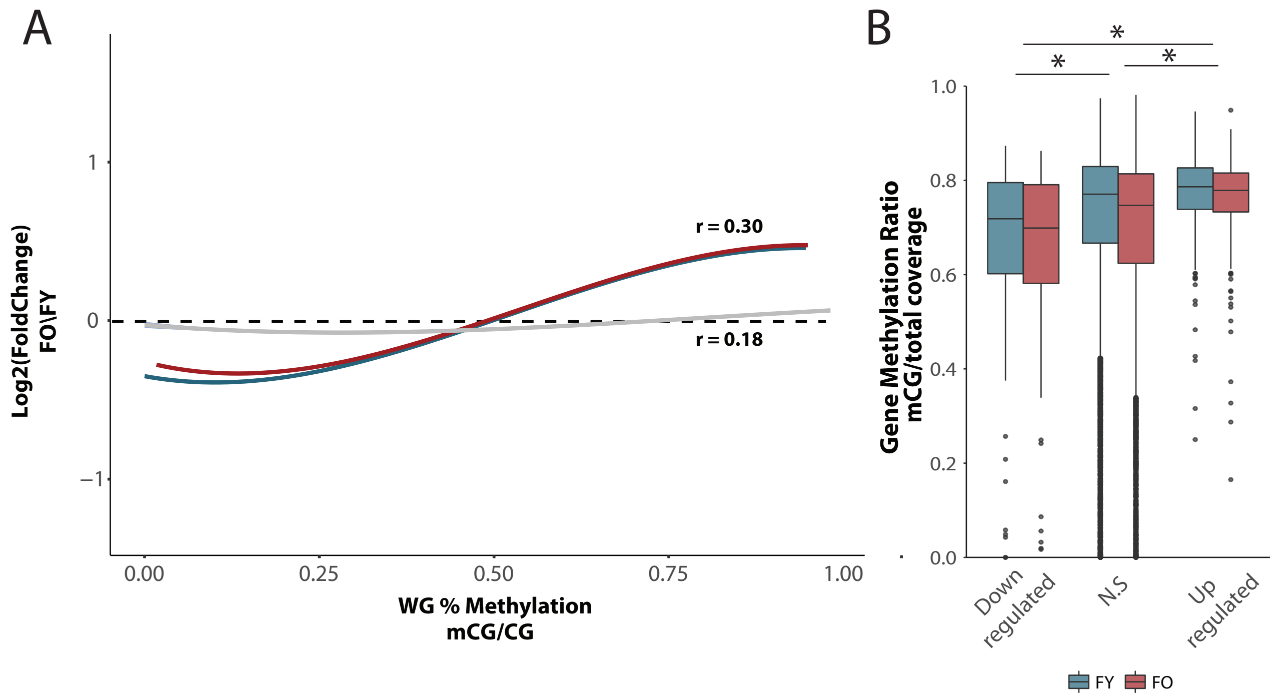

Supplemental Figure 3. Age-related differentially expressed genes are positively associated with gene body methylation in the Liver A) Genes downregulated with aging have lower gene body methylation at young age (blue regression line) compared to genes upregulated with aging in the liver. This relationship is maintained with aging (red regression line). Curve corresponds to the polynomial regression curve across significant (red and blue) and non-significant (black) differentially expressed genes. B) Box plot of whole gene methylation grouped by genes upregulated, non-differentially expressed, and downregulated genes in the liver. * $p < 0.001$ (Kruskal-Wallis Test).
