# Supplementary material for: Early-life DNA methylation profiles are indicative of age-related transcriptome changes
Source: Epigenetics Chromatin. 2019 Oct 8;12:58. doi: 10.1186/s13072-019-0306-5 (PMC6781367; doi:10.1186/s13072-019-0306-5)
Supplement: Supplementary file 10 — Additional file 10: Figure S6. Direction of change of age-related differentially expressed genes can be predicted based on DNA methylation profiles. Correlation matrices of different epigenetic features in downregulated genes with aging (A) and upregulated genes with aging (B). Area under the curve of the receive operating characteristics (ROC) curve showing the classification accuracy of age-related differentially expressed genes to upregulated and downregulated genes for Random Forest model in males (C) and females (D) trained based on baseline methylation and promoter and gene body DNA methylation. TRatio—gene ratio; wg—whole gene; tss—transcription start site; my—male young; mo—male old. [file 13072_2019_306_MOESM10_ESM.pdf]

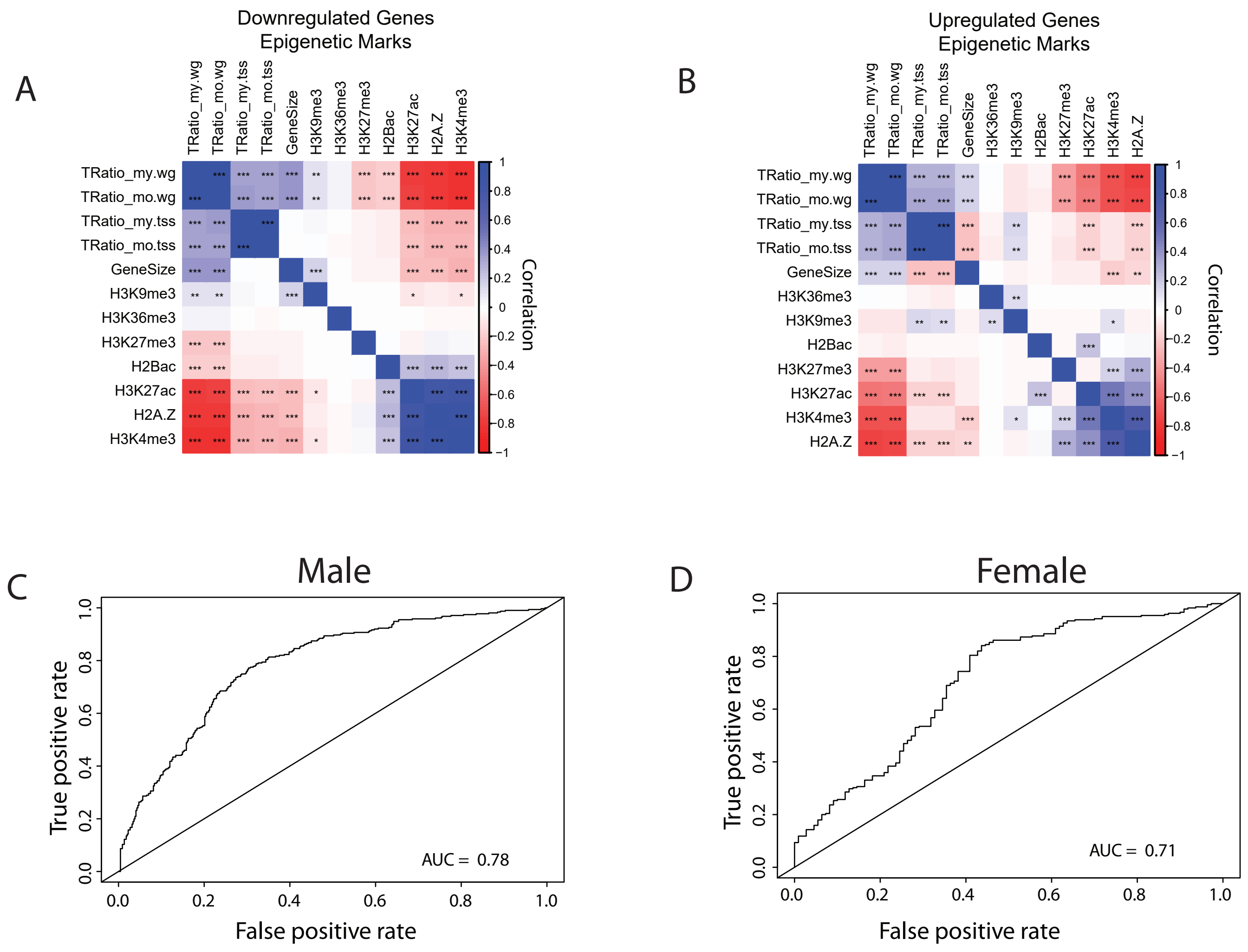

Supplemental Figure 4. Direction of change of age-related differentially expressed genes can be predicated based on DNA methylation profiles. Correlation matrices of different epigenetic features in downregulated genes with aging (A) and upregulated genes with aging (B). Area under the curve of the receive operating characteristic (ROC) curve showing the classification accuracy of age-related differentially expressed genes to upregulated and downregulated genes for Random Forest model in males (C) and females (D) trained based on baseline methylation and promoter and gene body DNA methylation. TRatio - gene ratio; wg - whole gene; tss - transcription start site; my - male young; mo - male old.
