# Supplementary material for: Early-life DNA methylation profiles are indicative of age-related transcriptome changes
Source: Epigenetics Chromatin. 2019 Oct 8;12:58. doi: 10.1186/s13072-019-0306-5 (PMC6781367; doi:10.1186/s13072-019-0306-5)
Supplement: Supplementary file 11 — Additional file 11: Figure S7. Sequencing alignment and differentially methylated region calling summary statistics. A. Boxplots representing the mapping efficiency per group. B. Overall genomic sequencing coverage. C. Boxplots showing the number of CpGs covered. D. Boxplots representing the average CpG coverage per group. E. Line plot of the number of DMRs mapped to genes (black and red) and gene promoters (green and blue) passed filtering from RNA-sequencing. F. Line plot showing the number of CpG in regions passed filtering for differential methylation. [file 13072_2019_306_MOESM11_ESM.pdf]

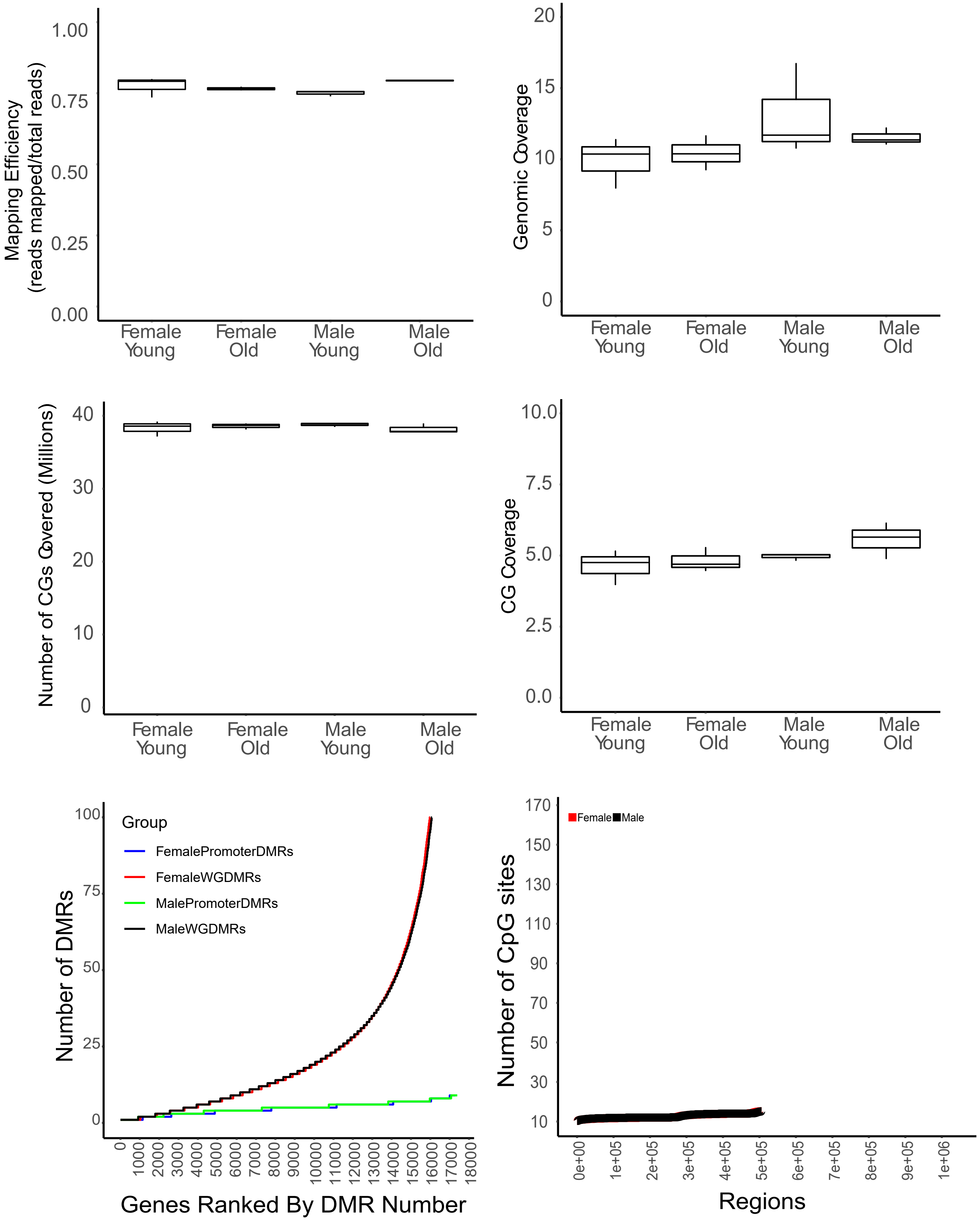

Supplemental Figure 6. Sequencing alignment and differentially methylated regioncalling summary statistics. A. boxplots representing the mapping efficiency per group. B. Overall genomic sequencing coverage. C. Boxplots showing the number of CpGs covered. D. boxplots represening the average CpG coverage per group. E. Line plot of the number of DMRs mapped to genes (black and red) and gene promoters (green and blue) passed filtering from RNA-sequencing. F. Line plot showing the number of CpG in regions passed filtering for differenitlal methylation.
